# News and Events of the Week

**Published:** 1911-08-12

**Authors:** 


### NEWS AND EVENTS OF THE WEEK.

The Winter Session of the Middlesex Hospital Medical School, 1911-12, will open on Monday, October 2, at 3 p.m. Dr. Berkeley will give the introductory address, after which the prizes will be distributed by Commendatore Marconi. The annual dinner takes place at the Trocadero the same evening at 7 o'clock precisely, with Dr. Wethered in the chair.

Owing to the extension of their business, Liebig's Extract of Meat Co., Ltd., proprietors of Lemco, Oxo, Fray Bentos canned goods, etc., are removing the whole of their offices this week to the handsome new building called "Thames House" which they have erected at Queen Street Place, London, E.C. The offices of Messrs. Corneille, David and Co. are also being removed to the same address.

During the last ten years the total numbers of recorded deaths from plague in India reached the enormous total of nearly seven millions. The highest plague mortality during the decennial period was reached in 1907, when the number of deaths was 1,315,892, and the lowest was in 1909, in which year the deaths numbered 178,808.

The Legislature of the State of New York has passed a Bill whereby some £13,500 have been appropriated for the erection and equipment of a hospital for the treatment of malignant diseases in connection with the Gratwick Laboratory, so that Buffalo is now to become a centre of scientific research. Every disease of a malignant character and of unknown origin will be studied there, though probably most of the work done will be confined to research with regard to cancer and allied conditions. The beds will be allocated to cases selected with a view to their being likely to derive benefit from the treatment provided, and not promiscuously to all suffering from the disease. The foundation is essentially to be a research institute, and patients will be admitted only on the understanding that they are to be wholly under the control of the staff and the treatments outlined by the staff.

